# Supplementary material for: Association of Self-Reported Physical Fitness during Late Pregnancy with Birth Outcomes and Oxytocin Administration during Labour—The GESTAFIT Project
Source: Int J Environ Res Public Health. 2021 Aug 3;18(15):8201. doi: 10.3390/ijerph18158201 (PMC8346096; doi:10.3390/ijerph18158201)
Supplement: Supplementary file 1 [file ijerph-18-08201-s001.zip › ijerph-1304763-supplementary.pdf]

Supplementary material

Table S1. Inclusion and exclusion criteria in the GESTAFIT project.

Inclusion criteria
- Pregnant women aged 25-40 years old with a normal pregnancy course.
- Answering "no" to all questions on the PARmed-X for pregnancy.
- Being able to walk without assistance.
- Being able to read and write properly.
- Informed consent: Being capable and willing to provide written consent.
*In addition, specific inclusion criteria for data analysis are: pregnancy with single foetus, spontaneous or instrumental vaginal birth, and caesarean without maternofoetal pathology (or any other indication that does not involve maternofoetal risk, such as disproportion, failed induction, no foetal progression or non-cephalic presentation).
Exclusion criteria
- Acute or terminal illness.
- Malnutrition.
- Inability to conduct tests for assessing physical fitness or exercise during pregnancy.
- Underweight.
- Pregnancy risk factors (such as hypertension, type 2 diabetes, etc.).
- Multiple pregnancy.
- Chromosopathy or foetal malformations.
- Uterine growth restriction.
- Foetal death.
- Upper or lower extremity fracture in the past 3 months.
- Presence of neuromuscular disease or drugs affecting neuromuscular function.
- Being registered in another exercise program.
- Doing more than 300 minutes of at least moderate physical activity per week.
- Unwillingness either to complete the study requirements or to be randomised into the control or intervention group.
